# The Impact of Peri-Implantitis on Systemic Diseases and Conditions: A Review of the Literature

**DOI:** 10.1155/2021/5536566

**Published:** 2021-05-15

**Authors:** Katherine Radaelli, Alice Alberti, Stefano Corbella, Luca Francetti

**Affiliations:** ^1^Department of Biomedical, Surgical and Dental Sciences, Università degli Studi di Milano, Milan, Italy; ^2^IRCCS Istituto Ortopedico Galeazzi, Milan, Italy; ^3^Department of Oral Surgery, Institute of Dentistry, I. M. Sechenov First Moscow State Medical University, Moscow, Russia

## Abstract

While periodontitis has been proven to have an impact on systemic conditions, such as cardiovascular diseases, pregnancy complications, or poor glycemic control in diabetic patients, the influence of peri-implantitis on systemic health has not been adequately explored in the literature as yet. The existing evidence suggests that peri-implant lesions lead to more intense inflammatory response than periodontitis. Given the analogies between periodontal diseases and peri-implantitis, the aim of the present paper was to review the scientific evidence about the potential correlation between peri-implantitis and systemic diseases and conditions. Two clinical trials on animals reported that experimental peri-implantitis determined an alteration in hematological and biological parameters. One human study explored the risk indicators for cardiovascular diseases and found that patients with peri-implantitis had significantly higher levels of triglyceride, uric acid, and white blood cells and lower levels of vitamin D. It was described in the literature that periodontitis affects cardiovascular health through a number of mechanisms, including the increase in systemic mediators of inflammation, which also has a role in the worsening of glycemic control in diabetic patients. Similarly, peri-implantitis may influence the systemic status through inflammatory cytokines such as IL-1, IL-6, and IL-10 and matrix metalloproteinases. One microbiological mechanism, based on the systemic dissemination of periodontal bacteria, has been hypothesized for cardiovascular diseases and pregnancy complications. Again, it is plausible that the same could occur in peri-implantitis. In conclusion, only few studies explored the systemic impact of peri-implantitis. Although changes in hematological parameters, biochemical parameters, and inflammatory markers have been reported in peri-implantitis, further studies are needed to investigate this correlation.

## 1. Introduction

The term peri-implantitis is used to define one local pathological condition affecting osseointegrated dental implants. It is characterized by the inflammation of the peri-implant tissues, showing bleeding on probing with or without suppuration, an increase in probing depth, and bone resorption, as evaluated through the periapical radiograph [1]. Peri-implantitis is a significantly prevalent disease, being observed in 1% to 47% of the subjects treated with implant-supported prosthesis [2] and in 1.1% to 85% of implants [3], as it was reported in systematic reviews of the literature. Considering long-term studies, one paper from our research group reported that, after 10 years from loading, the cumulative rate of implants free from peri-implantitis was 86.92% (60.69% patient level), in subjects treated with full-arch rehabilitations supported by four implants [4]. Another study reported an adjusted prevalence of peri-implantitis, ten years after prosthetic loading, of 38.4% patient level, based on a systematic review of the literature on a total of 15 studies [3]. The results are substantially coherent with those published before in another comprehensive review on the same topic [5].

Since peri-implantitis and peri-implant mucositis are extremely prevalent in the population, the importance of preventing the development of the diseases depends on identifying the risk factors actually associated to peri-implant diseases. The existing literature has highlighted the role of oral hygiene and, consequently, of the attendance on a strict maintenance program in reducing the risk of the development of peri-implantitis [6]. Another known risk factor for peri-implantitis is represented by a history of periodontitis. Substantial scientific evidence supports the role of periodontitis in determining an increase of the risk of incurring in peri-implantitis in patients affected [7]. One systematic review of the literature published in 2018, although reporting a significant heterogeneity among the 19 papers included, found that subjects with periodontitis were 2.15-fold more prone to develop peri-implantitis than healthy subjects [8]. These results were substantially confirmed by more recent studies on the same topic [9, 10]. The available literature about the role of smoking and diabetes as risk factors for peri-implantitis is substantially inconclusive and needs to be expanded [7]. With regard to bone resorption, a recent study by Rasperini et al. [11] showed that radiographic peri-implant bone loss around teeth is statistically significantly higher in periodontally compromised patients (PCP) who are also smokers, compared with nonsmoking PCP.

Due to the prevalence of peri-implant diseases and to the relevance of the correlation between oral diseases and systemic conditions, the present review of the literature aimed at exploring the potential link between peri-implantitis and extraoral conditions.

## 2. The Periodontal and Peri-Implant Inflammatory Lesions

The basis of the correlation between periodontitis and peri-implantitis consists in the analogies that we can find between the two diseases. Similar to what we know for periodontitis, peri-implantitis is not associated to a specific microbiome profile, even though a higher prevalence of some bacterial species (*A. actinomycetemcomitans* and *P. intermedia*) as compared to healthy implants has been reported in a review on studies that used PCR-based assessment, hybridization techniques, pyrosequencing, and transcriptomic analyses [12]. The microbiological pattern, analyzed with checkerboard DNA-DNA hybridization, real-time PCR, and sequencing/pyrosequencing, appeared not to be significantly different between teeth and implants, even considering the difference of the surface characteristics of both substrates and of the environment, in general [13]. However, some authors found that *P. gingivalis* and *F. nucleatum* were more significantly associated to periodontitis than to peri-implantitis, although the microbiome is not substantially different [14]. Peri-implant communities show less diversity than periodontal microbial communities [15]. One study analyzing adjacent peri-implant and periodontal microbiomes in states of health and disease found that 85% of individuals shared less than 8% of abundant species between teeth and implants [16]. One additional finding that alludes to the differences between periodontal and peri-implant microbiota is the resistance of the latter to antibiotic regimens that are efficacious against periodontitis: peri-implant communities include gram-positive bacteria that are also resistant to beta-lactam antibiotics [15]. Specific gram-positive taxa identified in human peri-implantitis, such as *S. mitis* and *S. oralis*, are known to produce low-affinity penicillin-binding proteins; these proteins are antibiotic-binding sites, acquired through gene transfer, that confer high resistance to beta-lactam antibiotics.

Considering the histopathological features of the periodontal and peri-implant lesions, the study by Carcuac and Berglundh published in 2014 examined 40 soft tissue biopsies, evaluating if a difference existed between the two lesions [17]. Interestingly, the relative proportion of inflammatory cells in both lesions is similar, with the exception of a predominance of neutrophils in peri-implantitis inflamed tissues as compared to periodontitis. Moreover, the total inflamed area is 4-fold larger when evaluating peri-implant tissues than in periodontal ones, and the cells themselves involved in the inflammation process (plasma cells, macrophages, and neutrophils) are significantly larger. All the above-described features of peri-implantitis lesions could be the representation of a more intense inflammatory response around dental implants than around teeth, and inflamed tissue is less “anatomically confined.” [17] Another paper confirmed such outcomes, also reporting that peri-implantitis lesions showed more neutrophils, macrophages, and other inflammatory cells than periodontitis lesions [18]. The microbiological and inflammatory pattern of peri-implantitis is the representation of a host immune response, which is characterized by the expression of proinflammatory mediators which are similar to those expressed by periodontitis, namely, IL-1*β*, IL-6, IL-17, and TNF-a, and mediators of osteolysis which are RANK, RANKL, Wnt5a and proteinase enzymes, MMP-2, MMP-9, and cathepsin K [19].

## 3. The Impact of Periodontitis on Systemic Conditions

A significant number of scientific papers were published over the last decades about the potential impact of periodontal diseases on the systemic status of the affected subjects [20, 21]. One report published in 2016 found that approximately one-third of all the studies in periodontology registered on international online registers were about periodontal medicine [21].

The systemic conditions that were most commonly correlated to periodontal diseases and whose relation was corroborated by sound scientific evidence are the wide category of cardiovascular diseases (CVDs), the complications related to pregnancy (namely, preterm birth and low birth weight), the effect on the glycemic control in patients with diabetes, and rheumatoid arthritis.

The role of periodontal diseases in determining an association with cardiovascular diseases was explained on the basis of a number of mechanisms [22]:

1. Microbiological mechanisms based on the possibility of periodontal bacteria to invade and infect distant tissues [23].
2. Inflammatory mechanisms that follow the increased systemic mediators of inflammation, including interleukins (IL-1, IL-4, IL-6, IL-8, and IL-18), C-reactive protein, matrix metalloproteinases, and other proteins such as galectin-3, whose salivary and serum levels were found to be higher in periodontal patients with or without coronary heart disease (CHD) than in subjects with CHD alone in a recent publication [24]. Increased levels of CVD biomarkers such as suPAR and galectin-1 have been recently associated to periodontal disease [25].
3. Increased thrombotic and hemostatic markers influencing inflammation.
4. Antibodies derived from the immune response to periodontal bacteria.
5. Other mechanisms involved in the pathogenesis of both periodontal and atherosclerotic diseases (virulence factors, extracellular reactive oxygen species, and dyslipidemia).
6. Treatment with specific cytokine inhibitors in patients with CVD is being considered and tested on preclinical models [26].

The plausibility of the association between CVD and periodontal diseases was confirmed by the efficacy of periodontal treatment in reducing, in general terms, the immune response markers, whose increase was observed in periodontal diseases [27].

Two mechanisms, one direct and one indirect, were hypothesized to be the fundamentals of the rationale of the correlation between periodontal diseases and adverse pregnancy outcomes [28]. The direct mechanism involves the invasion by oral microbes of the fetal-placental unit, disseminated by the hematogenous route; the indirect mechanism implies, as it happens for atherosclerotic diseases, that the inflammatory mediators, whose increase is induced by periodontitis, can increase the systemic inflammatory status, thus affecting the fetal-placental unit [28]. The mechanisms of association are proven in a number of studies, but the scientific evidence, derived from prospective studies, is still weak because of difficulties in controlling confounders and heterogeneity in study methods [29]. Moreover, the treatment of periodontal diseases is safe during pregnancy, but the effect in reducing the possibility of adverse pregnancy outcomes is still to be proved [29].

The inflammatory mechanisms are at the basis of the worsening of glycemic control in patients with both periodontitis and diabetes [30] as it appears that periodontitis can also augment the incidence of diabetes in one population [31]. A number of systematic reviews of the literature have highlighted the positive effects of periodontal treatment in improving the glycemic control in patients with diabetes [32, 33]. About that, the role of salivary matrix metalloproteinase (MMP-8) in relation to diabetes and periodontitis has been thoroughly investigated, and, in general terms, poor metabolic control is associated with increased salivary as well as circulating MMP-8 levels [34, 35]. Uncontrolled diabetes is related to increased risk of periodontitis, and it predisposes to accelerated periodontal destruction reflected in oral fluids as increased MMP-8 and -9 activation. Therefore, MMP-8 is an essential mediator in systemic subclinical inflammatory response in obesity and should be studied as a potential drug target [36].

In general terms, with lower scientific evidence, periodontal diseases were correlated to a number of other conditions, mainly by identifying an inflammatory common etiology: rheumatoid arthritis, Alzheimer's diseases, cancer, and others [37].

Given the analogies between periodontal diseases and peri-implantitis, in terms of microbiological profile and etiopathogenesis, and the known impact of periodontitis, and its consequences and factors, on a number of systemic diseases and conditions, the aim of the present narrative review of the existing literature was to explore the scientific evidence of the potential correlation between peri-implantitis and systemic diseases and conditions.

## 4. The Impact of Peri-Implantitis on Systemic Conditions

Due to the great heterogeneity of the results, it was impossible to perform any quantitative synthesis (meta-analysis) of the studies addressing the topic. Then, the assessment of the available literature in the field was performed narratively.

As it was discussed before, similar to periodontitis, peri-implantitis could be potentially correlated to systemic conditions regarding the onset mechanism and the immune response. In fact, a recent clinical trial on animals by Chaushu et al. [38] found that the presence of experimental peri-implant lesions could actually determine an alteration in hematological parameters. The authors investigated the correlation between peri-implant disease and anemia of chronic disease (ACD), which is a decrease of haemoglobin associated with chronic inflammatory diseases, by assessing complete blood count dynamics in experimental peri-implantitis in dogs. A significant increase in white blood cells, platelets, red blood cells, haemoglobin, and mean corpuscular haemoglobin concentration was found as a response to the local inflammation. After ligature removal and open flap debridement, these parameters returned to baseline values. This study demonstrated that intraoral disease and intervention determined a detectable systemic response and suggested a cytokine-mediated mechanism which is typical of ACD.

A second study by Chaushu et al. [39] explored the dynamics of serum biochemical parameters following experimental peri-implantitis in a dog model. The authors noted an increase in inflammatory parameters such as total protein and albumin concentrations.

Furthermore, another recent study [40] measured serum biochemical parameters that are known as CVD markers in patients affected by peri-implantitis, which resulted to show significantly higher levels of triglycerides, uric acid, and white blood cells as compared to healthy subjects or patients affected by peri-mucositis. They also had the lowest levels of vitamin D. The authors found a positive correlation between uric acid, triglyceride, and gingival index, pocket depth, bleeding on probing, and the amount of keratinized mucosa around the implants.

As it was observed for periodontitis, peri-implantitis may have a role in influencing the concentrations of all the molecules involved in the inflammatory process, triggering the host response towards the microbial contamination of the implant surface.

Many studies have examined the role of cytokines in patients with periodontitis [41–43]. Similarly, a recent review suggests that many inflammatory markers can be measured to diagnose peri-implant health and disease [44]. Indeed, the patterns of host osteo-immunoinflammatory modulation in patients with peri-implantitis involve specific biomarkers (including IL-1, IL-8, IL-10, and MMP-8) and could be helpful in the early diagnostic of the disease or to cooperate to prognostic information related to the status of the peri-implant breakdown. As described in detail in the previous section, the same cytokines were correlated to a number of systemic conditions and diseases, and the increase of their number could potentially involve a significant increase in the systemic inflammatory status.

**Figure 1:**
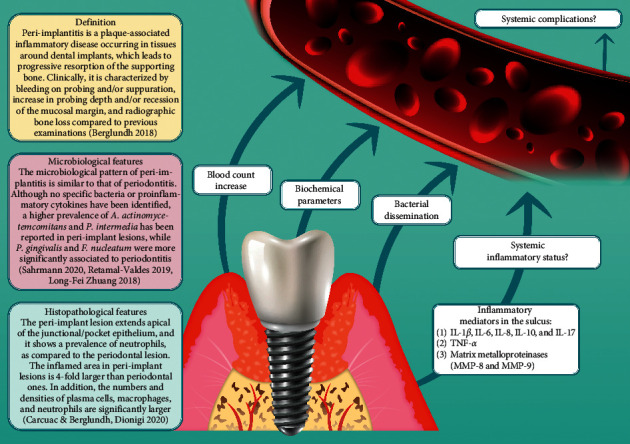
Different mechanisms of the potential impact of peri-implantitis on systemic health.

Interleukin-10 (IL-10), an anti-inflammatory cytokine produced by T-helper 2 cells, macrophages, and B cells, inhibiting the synthesis of proinflammatory cytokines such as IL-1, IL-2, IL-6, IL-8, TNF-*α*, and IFN-*γ* and acting as a B-cell stimulator, could play an important role in regulating cellular and humoral immune responses. Liskmann et al. [45] reported that a significantly higher concentration of IL-6 was found in saliva in the peri-implant disease group, while IL-10 could only be detected in patients with peri-implantitis, identifying a kind of specificity of such cytokines for the peri-implant disease. Moreover, the levels of IL-6 and IL-10 in the peri-implant disease group were positively correlated with clinical parameters. This result was later confirmed by Ata-Ali et al. [46] who found that IL-10, together with other cytokines (IL-1*β*, IL-6, and TNF-*α*), was significantly increased locally, in the sulcus of sites with peri-implantitis. These data suggest a significant relationship between proinflammatory cytokines and the inflammatory response in the peri-implant tissue, and these cytokines could be potentially valid diagnostic or prognostic markers of peri-implant tissue destruction. Bhavsar et al. [47] reported variations in the levels of three putative biological mediators (IL-1*β*, MMP-8, and MIP-1*α*) in peri-implantitis sites before and after surgical and antimicrobial therapy. IL-1*β* concentration in the peri-implant crevicular fluid could have diagnostic potential in peri-implantitis as its concentration was ten-fold higher in diseased sites as compared to healthy implants. Matrix metalloproteinase (MMP-8) plays a crucial role in the pathogenesis of periodontitis and is also a possible biomarker candidate in peri-implantitis. Thierbach et al. [48] investigated levels of MMP-8 in the peri-implant sulcus fluid and found that they increased in peri-implantitis-affected implants in both nonperiodontitis and periodontitis patients. After peri-implantitis treatment, a decrease in active MMP-8 in the peri-implant sulcus fluid was found in periodontitis patients.  Figure 1 summarizes different patterns through which peri-implantitis has been reported to affect the systemic status of the subject.

## 5. Conclusions

While the effect of periodontitis on systemic diseases, such as diabetes and pregnancy complications, has been extensively investigated and confirmed, there is a lack of evidence on the systemic effects of peri-implantitis. Similarly to periodontitis, peri-implantitis may have systemic effects. Only a limited number of studies investigated such correlation, finding that changes occur at a systemic level in patients affected by peri-implantitis, but the role of these changes and eventually their correlation with systemic pathologies still has to be explored. Moreover, the unfeasibility of any quantitative synthesis of the results, due to the great heterogeneity and the relatively low number of studies on this topic, affected the generalizability of our results.

The recent animal study by Chaushu et al. [38] found a correlation between peri-implant disease and alterations in complete blood count. Since ACD is considered to be mediated by cytokines that are produced as a consequence of the inflammatory process, we can hypothesize that peri-implant disease may affect blood count through a cytokine-mediated mechanism and speculate that a similar mechanism is plausible for other systemic conditions. Actually, serum inflammatory markers resulted higher in dogs with experimental peri-implantitis in another study by the same authors [39]. One recent human study reinforced this evidence, finding that common CVD markers such as triglycerides, uric acid, and white blood cells were significantly higher in patients with peri-implantitis [40].

A local increase in proinflammatory cytokines (e.g., IL-6 and IL-10) in the crevicular fluid has been reported for peri-implantitis, which may plausibly have systemic effects. Actually, it is conceivable that a mechanism similar to those occurring in periodontitis may be valid for peri-implantitis, also taking into account that peri-implantitis leads to a major immune response, as compared to periodontitis, being characterized by bigger lesions and more immune cells [17].

Starting from this assumption, the number of involved implants may also play a role in the magnitude of the influence that peri-implant diseases may have on systemic conditions.

In conclusion, peri-implantitis has been reported to induce systemic changes at different levels, including blood cell count, serum biochemical parameters, and cytokine levels, which may have an influence on systemic conditions and disease. However, it still remains to clarify if and how these might affect the onset or progression of systemic diseases. New studies with a prospective design are needed to explore such causal association.

## Data Availability

The data used to support the findings of this study are included within the article.
